# Clinical and pathophysiological aspects of type 1 autoimmune pancreatitis

**DOI:** 10.1007/s00535-018-1440-8

**Published:** 2018-02-19

**Authors:** Kazushige Uchida, Kazuichi Okazaki

**Affiliations:** Department of Gastroenterology and Hepatology, Kansai Medical University, 2-5-1 Shinmachi, Hirakata, Osaka 573-1010 Japan

**Keywords:** IgG4, Autoimmune pancreatitis, Regulatory T-cells, Regulatory B-cells, M2 macrophage, Basophil

## Abstract

In 1995, Yoshida and colleagues proposed the concept of “autoimmune pancreatitis” (AIP), which has recently been recognized as a new pancreatic inflammatory disease. Recent studies have suggested the existence of two subtypes of AIP: type 1, which involves immunoglobulin G4 (IgG4) and is the pancreatic manifestation of IgG4-related disease (IgG4-RD); and type 2, which is characterized by granulocytic epithelial lesions. Type 2 AIP is thought to be rare in Japan. Type 1 AIP is characterized by increased serum IgG4 concentrations, lymphoplasmacytic infiltrations, storiform fibrosis, and obliterative phlebitis. However, although type 1 AIP has become increasingly recognized, many clinical and basic issues remain to be solved. This review provides an overview of the recent clinical and basic knowledge of type 1 AIP.

## Introduction

Autoimmune pancreatitis (AIP) is a recently recognized new pancreatic inflammatory disease that is also recognized as a pancreatic manifestation of immunoglobulin-related disease (IgG4-RD). Recent research has described the clinical and pathophysiological features of type 1 AIP, but some details remain unclear. In this review, we discuss recent advances in type 1 AIP.

## History of AIP

A case of chronic pancreatitis with hypergammaglobulinemia and histologically inflammatory fibrosis was reported in 1961 by Sarles et al. [1]. This case report is thought to be the first report of AIP. Thirty years later, Kawaguchi et al. described histopathological findings characterized by lymphoplasmacytic infiltration, storiform fibrosis, and obliterative phlebitis as lymphoplasmacytic sclerosing pancreatitis (LPSP). The definition of LPSP provides the pathological basis of the disease that is now called type 1 AIP [2]. In 1995, Yoshida et al. proposed the concept of AIP [3]. In 2001, Hamano et al. reported that elevated serum immunoglobulin G4 (IgG4) levels were highly specific and sensitive for the diagnosis of AIP [4]. Thereafter, many investigators have reported on the clinical course and features of AIP, and it is now accepted as a new clinical entity of pancreatic inflammatory disorder [5–8]. In 2003, Kamisawa et al. suggested that AIP is a systemic disease that was an “IgG4-related autoimmune disease.” This suggestion was based on their findings that the pancreas and other involved organs showed abundant infiltration of IgG4-positive plasma cells and fibrosis [9]. Two other groups from Japan have also proposed that the possibility of the systemic disease involves IgG4. Yamamoto et al. proposed the term “IgG4-related plasmacytic syndrome” based on Mikulicz’s disease [10, 11]. Mikulicz’s disease was first reported in a case report by Johan Freisherr von Mikulicz-Radecki in 1892 [12]. This case report was discussed about 70 years ago from Sarle’s case report of chronic pancreatitis with hypergammaglobulinemia. In 2008, Masaki et al. proposed the term “IgG4-multiorgan lymphoproliferative syndrome” based on the presence of a lymphoproliferative disorder [13]. Although several concepts have been proposed, the Research Program for Intractable Disease of the Japan Ministry of Health, Labor, and Welfare unified these concepts in 2011 under the term as “IgG4-related disease,” which included type 1 AIP, IgG4-related sclerosing cholangitis, and Mikulicz’s disease, among others [14]. The term of IgG4-RD was also accepted at the first international symposium on IgG4-RD [15] (Table 1).

**Table 1:** History of autoimmune pancreatitis and IgG4-related disease

Year Name	Subjects	Refs.
1892 Mikulicz J	Mikulicz’s disease	[12]
1961 Sarles H et al.	Hypergammaglobulinemia in chronic pancreatitis	[1]
1991 Kawaguchi K et al.	Lymphoplasmacytic sclerosing pancreatitis	[2]
1995 Yoshida K et al.	Autoimmune pancreatitis	[3]
2001 Hamano H et al.	High serum IgG4 levels in sclerosing pancreatitis	[4]
2002 JPS	Diagnostic criteria for autoimmune pancreatitis	[21]
2003 Notohara K et al.	Idiopathic duct-centric pancreatitis	[16]
2003 Kamisawa T et al.	IgG4-associated autoimmune disease	[9]
2006 Yamamoto M et al.	IgG4-related plasmacytic syndrome	[10, 11]
2008 Masaki Y et al.	IgG4-multiorgan lymphoproliferative syndrome	[13]
2011 Shimosegawa T et al.	International Consensus Diagnostic Criteria for AIP	[18]
2011 Umehara H, et al.	IgG4-related disease	[14]
2011 Stone J	1st International Symposium on IgG4-RD	[15]
2012 JPS and RCIDP	Clinical diagnostic criteria of AIP 2011	[19]

*JPS* Japan Pancreas Society, *RIIDP* Research Committee of Intractable Diseases of the Pancreas

In terms of AIP, there have been reports of another unique histological pattern in the resected pancreata of patients with chronic mass-forming non-alcoholic pancreatitis with epithelial destruction by granulocytes (GEL) in Western countries [16, 17]. This histological pattern, which includes neutrophilic infiltration within the lumen and epithelium of the interlobular ducts, has been reported as idiopathic duct-centric pancreatitis (IDCP) by Notohara et al. [16]. Another name is AIP with granulocyte epithelial lesions (AIP with GEL) [17]. In 2011, the International Consensus Diagnostic Criteria for Autoimmune Pancreatitis (ICDC) proposed the classification of AIP into type 1 AIP (LPSP) and type 2 AIP (IDCP) [18]. In Japan, the clinical diagnostic criteria of AIP 2011 were proposed by the Japan Pancreas Society (JPS) and the Research Committee of Intractable Diseases of the Pancreas. The JPS 2011 is based on the ICDC and a simplified checklist of items for diagnose of type 1 AIP [19], because most Japanese AIP cases are type 1 AIP [20] (Table 2).

**Table 2:**
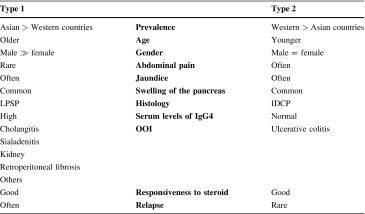
Characteristics of type1 and type2 autoimmune pancreatitis

*LPSP* lymphoplasmacytic sclerosing pancreatitis, *IDCP* idiopathic duct-centric pancreatitis, *OOI* other organ involvement

## Diagnosis of type 1 AIP

In 2002, the JPS first proposed the diagnostic criteria for AIP, which consist of three types of findings (i) image findings, such as irregular narrowing of the main pancreatic duct (MPD) (greater than one-third of the entire MPD) and pancreatic swelling; (ii) serological findings showing hypergammaglobulinemia (> 2 g/dL), elevation of serum IgG (> 1800 mg/dL), or autoantibodies; and (iii) characteristic pathological findings, including lymphoplasmacytic infiltration and fibrosis [21]. After the revision of the Japanese diagnostic criteria and the proposal of several new diagnostic criteria, we currently use two major sets of diagnostic criteria in Japan: ICDC as described above, and the clinical diagnostic criteria of AIP 2011 (JPS 2011), which were proposed by the JPS and the Research Committee of Intractable Diseases of the Pancreas supported by the Japanese Ministry of Health, Labor, and Welfare [19]. The ICDC correspond to the diagnostic methods of each country: and both type 1 and type 2 AIP can be diagnosed. For example, pancreatograms acquired using endoscopic retrograde cholangiopancreatography (ERCP) have traditionally been important in the diagnosis of AIP in Japan. In contrast, in Western countries, ERCP is not generally used for the diagnosis of AIP. In ICDC, type 1 AIP can be diagnosed by assessing a combination of five primary cardinal features: (i) imaging features of (a) pancreatic parenchyma [computed tomography (CT) or magnetic resonance imaging (MRI)] and (b) pancreatic duct [ERCP or magnetic resonance cholangiopancreatography (MRCP)]; (ii) serology (IgG4); (iii) other organ involvement; (iv) histopathology of the pancreas; and (v) response to steroid therapy. Furthermore, these cardinal features (i–iv) are divided into level 1 and level 2. On the other hand, the diagnosis of type 2 AIP is made by assessing a combination of four of the primary cardinal features from type 1, excluding serology (IgG4): (i) imaging features of (a) pancreatic parenchyma (CT/MRI) and (b) pancreatic duct (ERCP or MRCP); (ii) ulcerative colitis as other organ involvement; (iii) histopathology of the pancreas; and (iv) response to steroid therapy. Each criterion, except for steroid responsiveness, is classified as either level 1 or level 2 collateral criteria in a similar manner as used for type 1 AIP. Thus, many factors lead to the diagnosis in ICDC, which may have a complex presentation to a general gastroenterologist. Therefore, the proposal of JPS 2011 is based on the ICDC and a simplified checklist of items for diagnosis of type 1 AIP. The main characteristics of the JPS2011 are as follows: (i) in diffuse type type 1 AIP, ERCP is not essential; but in segmental/focal-type type 1 AIP, ERCP is still essential; (ii) serological findings in IgG4; (iii) other organ involvement (sclerosing cholangitis, sclerosing dacryoadenitis/sialoadenitis, retroperitoneal fibrosis) included clinically or histologically in diagnostic lists; (iv) resected pancreata can be used for diagnosis; and (v) a steroid trial is added as an optional item.

The accuracy of the existing diagnostic criteria has been investigated; the ICDC was found to be the most accurate among the available diagnostic criteria [22], and the sensitivities of the ICDC and JPS 2011 were 95.1, and 86.9%, respectively. The JPS 2011 is a set of diagnostic criteria for type 1 AIP. However, type 2 AIP also can be picked up as a possible diagnosis. The JPS 2011 requires ERP for the segmental/focal type of disease, but not for the typical diffuse type of AIP. A pancreatogram by ERP is useful for diagnosis [22, 23], but it has been reported that post-ERCP pancreatitis occurred in 1.1% of the patients in Japan [24].

The use of endoscopic ultrasound-fine-needle aspiration (EUS-FNA) procedures has been increasing and may eventually become common in Japan. However, two possible problems may arise with the spread of EUS-FNA due to the handling of small materials: IgG4-positive cells are detected in pancreatic cancer, and neutrophils infiltrate in the pancreas with type 1 AIP. There have been several recent reports of IgG4-positive cells associated with pancreatic ductal adenocarcinoma [25–27]. According to the comprehensive diagnostic criteria of IgG4-RD, Fukui et al. reported IgG4-positive cells in pancreatic ductal adenocarcinoma. The ratio of IgG4/IgG was > 40% in 43, 29, and 14% of the main cancer lesions, a non-cancerous lesion around the cancer, and an obstructive pancreatitis lesion, respectively [27]. In the comprehensive diagnostic criteria of IgG4-RD [14], there are two histopathological items: > 40% of IgG-positive plasma cells and > 10 IgG4-positive cells per high powered field (hpf) in samples. In this report, 89% of type 1 AIP cases showed an IgG4/IgG ratio > 40% and > 10 IgG4-positive cells per hpf. In 5% of pancreatic cancer cases, the main cancer lesion and obstructive pancreatitis lesion satisfies these two items related to the pathological features of the comprehensive diagnostic criteria of IgG4-RD [27].

Neutrophil infiltration is a characteristic finding in type 2 AIP. In general, it is thought that type 2 AIP is rare in Japan, but there are some reports on its diagnosis by EUS-FNA [28, 29]. It has been reported that there is no significant difference in neutrophil infiltration around the intralobular pancreatic ducts between type 1 and type 2 AIP have been found. Moreover, in one LPSP case, GELs were present in the intralobular pancreatic ducts [30]. These results show that an AIP diagnosis must be made carefully on the basis of the number of IgG4-positive plasma cells or infiltration of neutrophils as well as the presence or absence of GELs with a small biopsied sample obtained by EUS-FNA.

In the future, the JPS 2011 may be revised regarding the necessity of ERP for diagnosis of the focal/segmental type of type 1 AIP, the handling of EUS-FNA, and the validity of OOI and so on.

## Pathophysiology of type 1 AIP

### IgG4

IgG4 is the least amount of the four subclasses of IgGs; the immunoglobulin classes and subclasses are defined by the sequence of their heavy-chain constant domains. There are amino acid differences in the CH2 domain between IgG1 and IgG4 that lead to weak or negligible binding of IgG4 to both C1q and Fcγ receptors [31, 32]. Especially, a unique feature of IgG4 is its ability to form “half-antibodies” through the Fab-arms exchange by swapping a heavy-chain and attached light chain (Fab-arm exchange) [33]. The amino acid variation at the hinge region of IgG4 forms asymmetric antibodies that consist of half-antibody fragments. This asymmetric IgG4 can recognize two different antigens. Asymmetric IgG4 is unable to crosslink antigens to form immune complexes. Therefore, the lack of immune complex formation and the low affinity for the C1q and Fc receptor might be responsible for the anti-inflammatory function.

Autoantibodies, including autoantibodies to lactoferrin, carbonic anhydrase II, and pancreatic trypsin inhibitor, have been reported in patients with type 1 AIP [8]. However, IgG4-type autoantibodies have not been detected in the patients with type 1 AIP.

## Acquired immune system

### T-cells

Recent studies have suggested possible multi-pathogenic factors in the development of type 1 AIP. However, the pathogenic mechanism of type 1 AIP remains unclear. From the viewpoint of acquired immunity, the Th1/Th2 immune balance is an important consideration. In IgG4-RD (include type 1 AIP), Th2 type immune balance has an important role in the pathogenesis of IgG4-RD. In addition, IgG4-RD is associated with abundant infiltration of regulatory T-cells (Tregs) into target organs. The cytokine profile of IgG4-RD reportedly includes Th2 cytokines (IL-4, IL-5, and IL-13) and regulatory cytokines (IL-10 and TGF-β) [34–37]. In terms of Tregs, circulatory naïve (CD4^+^CD25^+^CD45RA^+^) Tregs are significantly decreased, whereas CD4^+^CD25^high^ and memory Tregs are significantly increased in the peripheral blood of patients with type 1 AIP. Increased peripheral Tregs are positively correlated with serum levels of IgG4 [38]. In addition, increased quantities of inducible costimulator (ICOS)-positive Tregs may influence IgG4 production via IL-10 in type 1 AIP, and ICOS-negative Tregs may influence fibrosis via TGF-β [39]. Production of IgG4 may reflect over expression of anti-inflammatory cytokines, such as IL-10. These findings suggest that IgG4 does not act as a pathogenic factor, nor is it an anti-inflammatory factor in type 1 AIP. Further studies are necessary to clarify the precise role of IgG4 in IgG4-RD and include type 1 AIP.

### B-cells

Regulatory B-cells (Bregs) have been reported to appear with several surface markers. Sumimoto et al. reported that CD19^+^CD24^+^CD38^high^ Bregs increased, whereas CD19^+^CD24^high^CD27^+^ Bregs decreased in type 1 AIP [40]. These data indicate that CD19^+^CD24^high^CD38^high^ Bregs seemed to increase reactively to suppress the disease activity, and CD19^+^CD24^high^CD27^+^ Bregs might be involved in the development of type 1 AIP. Recently, it was reported that plasmablasts may have an important role in IgG4-RD [41]. When considered in the context of the effectiveness of rituximab [42], the role of B-cells in type 1 AIP must be clarified.

## Innate immune system

Yanagawa et al. reported that Toll-like receptors (TLR)2 or TLR4-positive basophils infiltrated into the pancreas of patients with type 1 AIP and that the ratios of basophils activated by TLR4 stimulation in type 1 AIP and atopic dermatitis were significantly higher than those in healthy subjects [43]. Watanabe et al. reported that TLRs and nucleotide-binding oligomerization domain-like receptors activation in the monocytes [44] and basophils [45] of patients with IgG4-RD enhanced IgG4 production by B-cells from healthy control individuals via production of B-cell-activating factor (BAFF). Moreover, Fukui et al. reported that abundant infiltration of TLR-7-positive M2-macrophages was observed in the resected pancreata of patients with type 1 AIP [46]. Activated basophils may lead to the differentiation of inflammatory monocytes into M2 macrophages, and influence the Th2 immune environment and may also affect the production of IgG4 via TLR signaling.

Previously, neutrophils have been shown to infiltrate type 1 AIP [30], because IL-8 expressed in the pancreatic duct epithelia in type 1 and type 2 AIP [30]. They also reported that significantly increased neutrophil infiltration around the interlobular pancreatic duct in type 2 AIP might depend on secretion of granulocyte chemotactic protein-2 [30]. In addition, Arai et al. studied the relationship between neutrophil extracellular traps (NETs) and IgG4 production in type 1 AIP. They found that the pancreata of patients with type 1 AIP but not those of the controls contained NETs. In the presence of NETs, plasmacytoid dendritic cells produced IFN-α and BAFF and induced the control of B-cells to produce IgG4 [47]. Thus, these findings suggested that an innate immune response is involved in the development of type 1 AIP.

## Hypothesis of pathophysiology of type 1 AIP

**Fig. 1:**
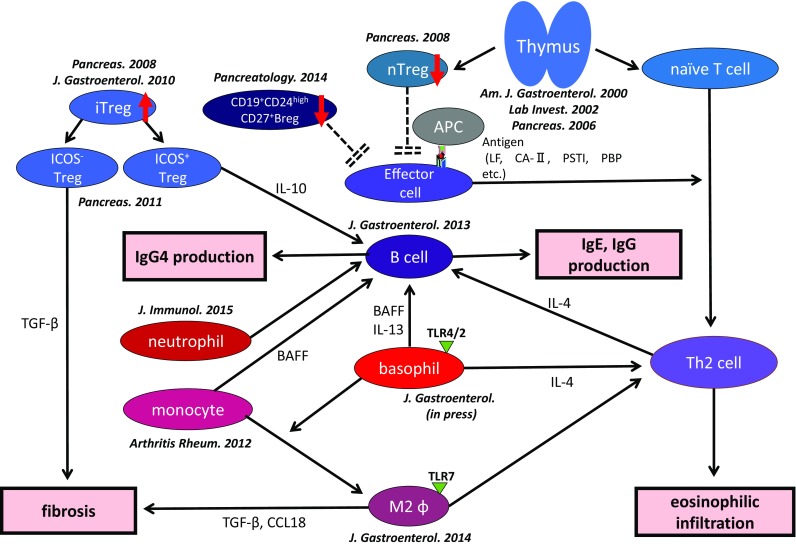
Proposal for the pathophysiology of type 1 AIP. Decreased numbers of naïve regulatory T-cells and CD19^+^CD24^high^CD27^+^ regulatory B-cells (Bregs) may be involved in the induction of type 1 AIP. Inducible regulatory T-cells (iTregs) and CD19^+^CD24^+^CD38^high^ Bregs increased reactively. The progression of the disease was supported by an increased Th2 immune response. The production of IgG4 may be regulated by IL-10 secreted from ICOS-positive Tregs, and Basophils and monocytes also regulate the production of IgG4 via TLR- and NOD-like receptor signaling. Fibrosis may be regulated by TGF-β secreted from ICOS-negative Tregs and M2 macrophages. M2 macrophages may also contribute to the Th2 immune response in type 1 AIP. Neutrophils also influence IgG4 production via NETs

We suggest the following pathophysiology of type 1 AIP. In the initial stage of type 1 AIP, because of decreased naïve Tregs and CD19^+^CD24^high^CD27^+^ Bregs, effector T-cells are involved in the tissue damage. IL-10 and TGF-ß from increased inducible Tregs induce the switch from B-cells to IgG4-producing plasma cells and fibrosis, respectively. Basophils lead to differentiation of inflammatory monocytes into M2 macrophages, affect production of IgG4 via TLR signaling, and influence the Th2 immune environment. M2 macrophages also contribute to the fibrosis and Th2 immune reaction. Neutrophils also influence IgG4 production via NETs (Fig. 1).

## Treatment for type 1 AIP

The recommended the first-line treatment for type 1 AIP is steroid therapy, because of the observed good response. The rapid response to glucocorticoids is one of the primary characteristics of type 1 AIP. A poor response to steroid therapy might indicate misdiagnosis, especially misdiagnosis in the case of pancreatic cancer. The Japanese consensus guidelines have proposed the recommended initial oral prednisolone dose for induction of remission to be 0.6 mg/kg/day, which is administered for 2–4 weeks. The dose is then tapered by 5 mg every 1–2 weeks to a maintenance dose (5.0–7.5 mg/day) that should be continued for 3 years as a maintenance therapy [48]. In Japan, to prevent relapses in type 1 AIP, many patients are advised to continue daily low-dose prednisolone for months to years following induction of remission. However, this maintenance of steroid therapy has both advantages and disadvantages. In Western countries, steroid treatment is often limited to a short-term therapy because of ongoing concerns about the risks of adverse events, such as diabetes mellitus, osteoporosis, cataracts, peptic ulcers, and infections [49]. A multicenter study in Japan reported that relapse occurred significantly less often during maintenance steroid therapy (23%) than after discontinuation of therapy (34%) [50]. Recently, the outcome of a Japanese randomized-controlled study regarding maintenance steroid therapy was reported [51]. This report provided evidence to support the usefulness of maintenance steroid therapy.

Unfortunately, despite the high initial remission rates, 15–60% of patients will develop disease relapse either after cessation of steroid therapy or during the weaning of the steroid dose [17, 52, 53]. In most cases of relapsed type 1 AIP, re-administration or an increased dosage of prednisolone is effective. In Western countries, there have been reports of concomitant use of immunomodulatory drugs, such as azathioprine, methotrexate, and mycophenolate mofetil, for patients with type 1 AIP who relapsed or were resistant to steroid therapy [52, 54, 55]. It has been reported that relapse-free survival was similar in patients treated with steroids plus immunomodulatory drugs compared to that in patients treated with steroids alone, and nearly half of the patients on immunomodulatory drugs will relapsed during treatment at the Mayo clinic [56]. On the other hand, an Italian group recently reported the efficacy of AZA as a maintenance therapy to prevent disease relapse in AIP [57]. They concluded that AZA was an effective and safe treatment to prevent AIP relapses.

Rituximab, a monoclonal anti-CD20 antibody, has also been successfully used to treat IgG4-RD [42, 56]. A clinical trial that evaluated rituximab for the treatment of IgG4-RD showed efficacy even without concomitant glucocorticoid therapy [58]. B-cell depletion may be effective for type 1 AIP because of its powerful association with the pathogenesis. Rituximab is not yet approved for use in Japan, but it remains necessary to establish a second-line therapy that includes immunomodulatory drugs for the patients who relapse with type 1 AIP.

## Prognosis of type 1 AIP

Steroid therapy has been reported to improve pancreatic exocrine and endocrine function by reducing inflammation, fibrosis, and regeneration through correct aberrant cystic fibrosis transmembrane conductance regulator localization in the duct and to regenerate acinar cells in type 1 AIP [59]. These results indicate that the short-term prognosis of type 1 AIP is good.

The long-term prognosis, however, is not clear, because there are many unknown factors, including relapse, pancreatic exocrine or endocrine dysfunction, and associated malignancies that include pancreatic cancer. It is thought that approximately 10% (7–40%) of the patients with type 1 AIP develop pancreatic calcification or chronic pancreatitis [20, 60–64]. Maruyama et al. reported pancreatic head swelling and non-narrowing MPD in the pancreatic body as risk factors for the development of chronic pancreatitis in type 1 AIP [63, 64]. Narrowing of both Wirsung’s and Santorini’s ducts by pancreatic head swelling causes pancreatic juice stasis in the upstream pancreatic duct. Moreover, pancreatic juice stasis results in increased intra-pancreatic duct pressure that is resistant to MPD narrowing of typical type 1 AIP in the pancreatic body, which leads to MPD non-narrowing in this region.

Chronic pancreatitis has been reported as one of the risk factors for pancreatic cancer [65]. Ikeura et al. reported that patients with type 1 AIP had a higher risk of pancreatic cancer, similar to that of patients with ordinary chronic pancreatitis [66]. Shiokawa et al. reported that the risk of developing various cancers was highest during the first year after AIP diagnosis and speculated that AIP may be a manifestation of paraneoplastic syndrome. It remains unclear whether there is a definitive risk factor for malignancy [67]. However, the risk of patients with type 1 AIP to develop cancer is a very important consideration.

## Conclusion

Type 1 AIP is now recognized as a pancreatic lesion of IgG4-RD. However, many clinical and basic issues still remain unclear in cases of type 1 AIP Unclear issues include its clinical features, diagnosis, treatment, prognosis, and pathogenesis of type 1 AIP. We believe that this article provides a foundation for clarifying a number of these issues.

## Abbreviations

AIP: Autoimmune pancreatitis
IgG4: Immunoglobulin G4
LPSP: Lymphoplasmacytic sclerosing pancreatitis
IDCP: Idiopathic duct-centric pancreatitis
GEL: Granulocytic epithelial lesion
ERCP: Endoscopic retrograde cholangiopancreatography
EUS-FNA: Endoscopic ultrasound-guided fine needle aspiration
TLR: Toll-like receptor
NOD: Nucleotide-binding oligomerization domain
ICOS: Inducible costimulator
Tregs: Regulatory T-cells
Bregs: Regulatory B-cells
